# The Incidence of Screw Failure in Fenestrated Polyaxial Pedicle Screws vs. Conventional Pedicle Screws in the Treatment of Adolescent Idiopathic Scoliosis (AIS)

**DOI:** 10.3390/jcm13061760

**Published:** 2024-03-19

**Authors:** Calogero Velluto, Michele Inverso, Maria Ilaria Borruto, Andrea Perna, Guido Bocchino, Davide Messina, Luca Proietti

**Affiliations:** 1 Department of Aging, Orthopaedic and Rheumatological Sciences, Fondazione Policlinico Universitario Agostino Gemelli IRCCS, 00168 Rome, Italy; michele.inverso01@icatt.it (M.I.); mariailaria.borruto01@icatt.it (M.I.B.); guido.bocchino01@icatt.it (G.B.); davide.messina01@icatt.it (D.M.); luca.proietti@policlinicogemelli.it (L.P.); 2 Department of Orthopaedics and Traumatology, Fondazione Casa Sollievo della Sofferenza IRCCS, 71013 San Giovanni Rotondo, Italy

**Keywords:** adolescent idiopathic scoliosis, posterior fusion, pedicle screw, hardware failure

## Abstract

**Background**: Adolescent idiopathic scoliosis (AIS) is a spinal pathology affecting 0.47–5.2% of the population, often requiring surgical intervention to control deformity progression. Posterior spinal instrumentation and fusion with pedicle screw fixation are standard procedures for AIS curve correction; however, implant failure remains a significant complication, especially in multi-level fusions. This retrospective cohort study aims to compare the failure rates between conventional pedicle screws (CPSs) and fenestrated pedicle screws (FPSs) in AIS treatment, with a focus on investigating potential causes of these failures. **Methods**: This study, conducted from January 2016 to December 2020, involves a two-center retrospective analysis of AIS patients undergoing posterior instrumented fusion. **Results**: Data from a total of 162 patients (122 females and 40 males) revealed a mean age of 14.95 years (range: 11–18). The CPS group consisted of 80 patients (56 females and 24 males), whereas the FPS group consisted of 82 patients (66 females and 16 males) stratified by Risser grade and Lenke Classification. Radiological assessments, clinical outcomes, and SRS-22 scores were evaluated pre-operatively, at 6 months, and post-operatively (minimum follow-up of 2 years). **Conclusions**: Fenestrated pedicle screws (FPSs) pose concerns due to their lower mechanical strength compared to solid screws. Understanding their limitations and optimizing their application in AIS treatment is essential.

## 1. Introduction

Adolescent idiopathic scoliosis (AIS) is a pathology with an overall prevalence of 0.47–5.2% in the current literature [1], which requires surgery only in 0.7% of cases in order to stop the progression of the deformity [2]. Posterior spinal instrumentation and fusion with pedicle screw fixation is the gold standard for AIS curve correction [3]. The goal of fusion is to stabilize the spine after the correction of deformities with the aim of achieving mechanical stabilization that guarantees biological fusion. To perform this surgery, the spinal implant must withstand compressive, torsional, and bending loads. For this reason, one of the most common complications in spinal fusion surgeries is implant failure, especially for multi-level fusion [4]. The success of fusion surgery relies heavily on the durability and stability of the spinal implant system, which must withstand various loads and stresses post-operatively.

Pedicle screws are widely used in deformity correction surgery, which remains highly challenging because of the technically demanding procedure involved with the potential for significant neurologic, vascular, and visceral injuries within the thoracolumbar region. Moreover, in severe deformities, the apical region is generally the portion with more stiffness where the positioning of the screw is also more challenging. In fact, the axial rotation of the vertebrae does not allow the surgeon to easily find the correct entry point in this region, especially on the concave side, where the pedicles are thinner, and so, transpedicular screw fixation is not always possible. For this reason, a fenestrated screw system could be used in which the guide wire prevents screw migration during insertion [5].

This system is an alternative design to conventional pedicle screws (CPSs), with it developed to be applied in minimally invasive or navigation techniques and inserted following the guidance wire along the planned path [6].

Although a 3.0 to 12.4% failure rate for conventional pedicle screws is reported in the literature [7], the prevalence of fenestrated pedicle screw (FPS) rupture remains unclear.

The purpose of this retrospective cohort study is to compare the rate of screw failure of CPSs and FPSs in the treatment of AIS and discuss the potential causes of these failures.

## 2. Materials and Methods

The present study is a retrospective radiographic and clinical analysis of patients affected by AIS who underwent posterior instrumented fusion and correction of deformities.

The study consists of a two-center retrospective investigation, collecting data from the institutional picture archiving and communication system (PACS), in a time range of January 2016 to December 2020. Patients’ deformities were classified according to the Lenke Classification [8]. The normality of the data was assessed using the Shapiro–Wilk test in IBM SPSS Statistics version 27.0. The Shapiro–Wilk test indicated that the data were normally distributed (*p* > 0.05).

### 2.1. Inclusion and Exclusion Criteria

Only cases with complete clinical and radiological data, pre-collected informed consent for scientific investigations, and a minimum follow-up of 24 months were considered for eligibility. The inclusion criteria were age between 10 and 18 years, a coronal Cobb angle ≤ 80°, a posterior-only approach, only pedicle screw instrumentation, the absence of thoracoplasty, and a minimum follow-up of 2 years. The exclusion criteria were congenital or neuromuscular scoliosis, previous spinal surgery, hook or wire use, post-operative infections, neoplastic diseases, and a Cobb angle > 81°.

### 2.2. Screw Design

**Figure 1:**
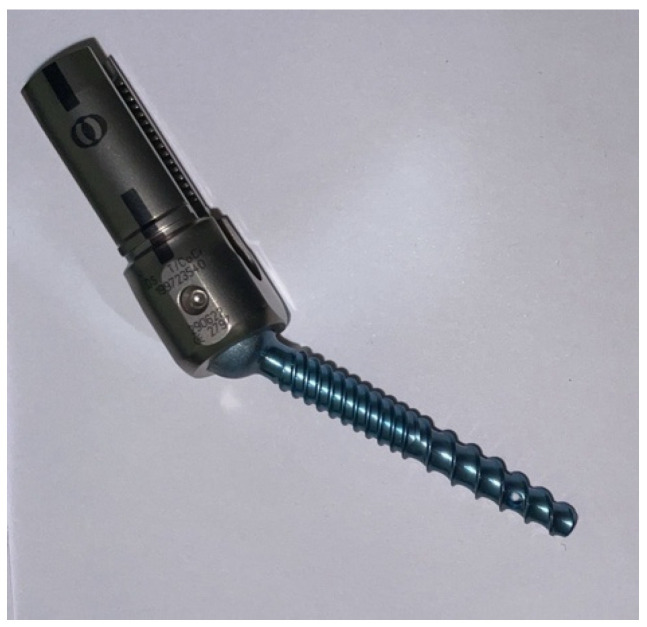
Example of a fenestrated pedicle screw (FPS) (Verse Depuy Cortical Fix, DePuy Spine Johnson & Johnson Co., Paramount Drive, Raynham, MA, USA).

The FPS used is a polyaxial, dual-lead threaded, and fully cannulated screw with three fenestrations in the distal tip (Verse Depuy Cortical Fix, DePuy Spine Johnson & Johnson Co., Paramount Drive, Raynham, MA, USA). The polyaxial screw head is designed to simplify the procedure for fitting rods. The dual-lead thread can provide different fixation requirements for the cortical and spongy bone. Cannulation of 1.75 mm allows for the injection of cement through a screw for indicated patients (Figure 1).

The CPS used is a polyaxial, double-lead thread screw (Expedium Verse Depuy, DePuy Spine Johnson & Johnson Co., Paramount Drive, Raynham, MA, USA) with a self-tapping and self-centering shank.

### 2.3. Surgical Techniques

All patients in Group A were positioned with conventional pedicle screws (CPSs), while all patients in Group B were positioned with fenestrated pedicle screws (FPSs) with the following surgical techniques.

#### 2.3.1. CPS Group

All of the patients underwent posterior surgery under general anesthesia with spinal cord monitoring of somatosensory and motor-evoked potentials. The patients were placed in the prone position on a radiolucent table. After a posterior midline incision, the subperiosteal dissection of the posterior soft tissue was performed. In all patients, the last two inferior vertebrae were instrumented on both the concave and convex sides to obtain a solid basal square; the immediate superior vertebra to the last two inferior ones was left without anchor points and then, every level was instrumented alternatively on the concave and convex sides of the curve, until the penultimate vertebra in the superior part, which was left without anchor points; the last superior vertebra was instrumented both in the concave and convex sides. The apical vertebra was always included in the instrumented vertebrae and instrumented only on the concave side. This construct, having strategic anchor points (apical vertebrae, terminal vertebrae, and alternative anchors on the curve extensions), allows one to perform a global correction of the curves. The laminae were thoroughly decorticated, the spinous process and the other spine constraints were removed in order to facilitate the correction maneuvers, and the bone graft obtained from decortication was used for fusion. Correction maneuvers implied the insertion of the rod in the concave side of the main curve as the first step, previously contoured in the sagittal profile of the instrumented segment. Generally, in order to obtain a balanced spine in the sagittal profile and to prevent the remodeling of the rod during correction, hyperkyphosis and lordosis were given to the pre-bent rod. The first step of correction was carried out by reducing the rod into the reduction tabs using the set screws in order to reach the screw head. This way, a segmental translation of the spine to the rod was obtained. After the rod was engaged in all anchors, the rod rotation instruments were attached to the rod, and the surgeon, together with the assistant, performed a global de-rotation of approximately 90° in the direction of the concave side. This maneuver allows for achieving the best correction. At the end of the correction maneuvers, the rods were observed inside and always connected using two transverse connectors.

#### 2.3.2. FPS Group

The same surgical technique was employed as previously described for patients who received fenestrated pedicle screws (FPSs). The key difference lies in the placement of the fenestrated screws. For conventional pedicle screws (CPSs), the procedure involves the use of a drill to initiate the placement, a gear shift to access the bone spongiosa, using a probe to ensure correct positioning, and then proceeding with screw implantation. For fenestrated pedicle screws (FPSs), a K-wire is inserted into all pedicles following palpation, a fluoroscopic examination is performed, and only then are the screws positioned.

### 2.4. Clinical Outcomes

Clinical outcomes were determined using the SRS-22 score [9]. Clinical status was evaluated pre-operatively, at 12 months after surgery, and at 24 months after surgery, using a ten-point itemized visual analog scale (VAS) for pain, mental health (MH), self-image (SI), function, and satisfaction. Intraoperative and post-operative complications were recorded.

### 2.5. Radiological Assessment

Pre-operative, early post-operative (6 ± 2 months FU), and post-operative (minimum follow-up 2 years ± 3 months) X-ray images were retrieved and reviewed using a dedicated workstation (Advantage Windows Workstation; GE Medical Systems, Milwaukee, WI, USA). The X-ray images were independently evaluated by three authors and a senior spinal surgeon (L.P). All patients were evaluated with anteroposterior (AP), lateral full-length standing, and lateral bending radiographs.

### 2.6. Statistical Analysis

Data are reported as means and standard deviations (SD). The normality of the data was tested. Categorical variables were compared using a two-tailed Fisher’s exact test, whereas continuous variables were compared using *t*-tests. Interrater reliability (IRR) between the three evaluators was calculated using Fleiss’ kappa statistic. A significance level of 0.05 was set for statistical significance. SPSS (Statistical Package for the Social Sciences) software 26, developed by IBM, Chicago, IL, USA, was used for data analysis.

## 3. Results

### 3.1. Patients Identification

Data from 162 patients (40 males and 122 females) who met the inclusion criteria were retrospectively analyzed. The patients were divided into two groups considering screw type. Group FPS and Group CPS. In Group CPS, the data from 80 patients (56 females and 24 males), with a mean age of 14.7 (range 11–18), were reviewed. Patients were stratified for Risser grade and Lenke Classification as reported in Table 1. The number of patients who underwent surgery using FPSs was 82 (66 females and 16 males). The mean age was 15.2 (range 11–18). Patients were stratified for Risser grade and Lenke Classification as reported in Table 1.

### 3.2. Radiological Findings

A summary of the main radiographic results can be found in Table 2. The mean follow-up duration was 3.9 years (ranging from a minimum of 2 to a maximum of 5 years). Sagittal curves did not show significant changes at any observational time point (Table 2).

All intra- and immediate post-operative data are presented in Table 3, classified based on the type of screws used: group A (CPS) and group B (FPS).

Radiographic analysis revealed a mean pre-operative Cobb value of 59.75°, which decreased to 20° immediately post-operatively (*p* = 0.001) and to 19.1° at the 2-year minimum follow-up (*p* = 0.003). The percentage of correction did not exhibit a statistically significant correlation with pedicle screw density or the type of screw used (CPS or FPS).

At the 2-year minimum follow-up, there were no statistically significant losses observed in the major structural curve correction among patients treated with either a CPS or an FPS.

The corrections of coronal curves expressed as apical vertebra rotation (AVR) were significant immediately post-operatively (*p* = 0.004). This parameter did not significantly vary when comparing the immediate post-operative time and the 2-year minimum follow-up.

At the 2-year follow-up, no significant difference was detected in terms of mean correction between the first group (CPS), which was 15.4%, and the second group (FPS), 15.7% (*p* > 0.05).

### 3.3. Clinical Outcomes

**Figure 2:**
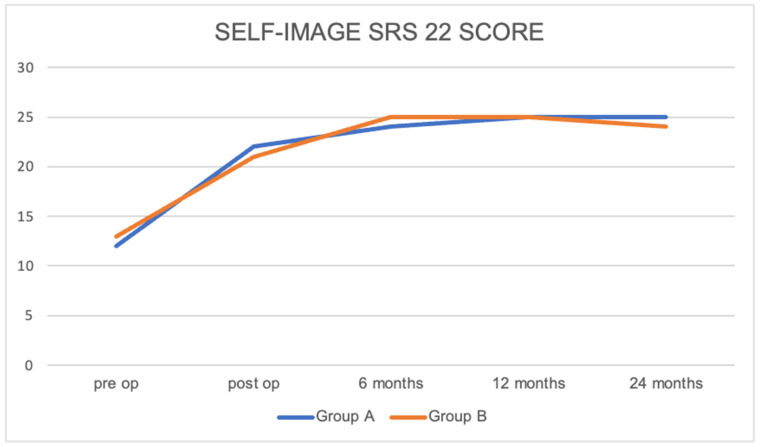
Difference in clinical outcomes between the two self-image sub-groups, not statistically significant (*p* > 0.05).

The SRS-22 test was used for the evaluation of the clinical outcome of each subgroup. At 6 months, a relevant improvement was demonstrated, with further significant progress observed between the 6-month and 2-year minimum follow-up. Self-image results revealed a notable improvement between the pre-operative period and 6 months. However, no significant difference was observed, even when considering the extreme point of the percentage correction range. The difference between the two self-image sub-groups (A versus B) was not statistically significant, 3.8 ± 0.52 vs. 3.9 ± 0.48 (*p* > 0.05), as shown in Figure 2.

There was a significant improvement in pain scores from baseline to the short-term follow-up period for both CPSs (Group A) and FPSs (Group B), with mean reductions of 3.8 ± 1.0 points and 3.5 ± 1.2, respectively (*p* < 0.05). Similarly, regarding function scores, both groups exhibited significant improvement from baseline to the short-term assessment, with mean increases of 4.2 ± 1.5 and 4.0 ± 1.3 points for FPSs and CPSs, respectively (*p* < 0.05). These improvements were sustained in the medium-term follow-up, with further reductions in pain scores of 1.8 ± 0.9 and 2.0 ± 0.8 points and additional increases in function scores of 2.5 ± 1.0 and 2.3 ± 0.9 points for FPSs and CPSs, respectively (*p* < 0.05). Despite these notable improvements, stratification based on Risser or Lenke grading did not reveal any significant differences in pain and function scores between the two screw types at any time point, suggesting comparable efficacy in the short and medium term.

### 3.4. Complications

**Figure 3:**
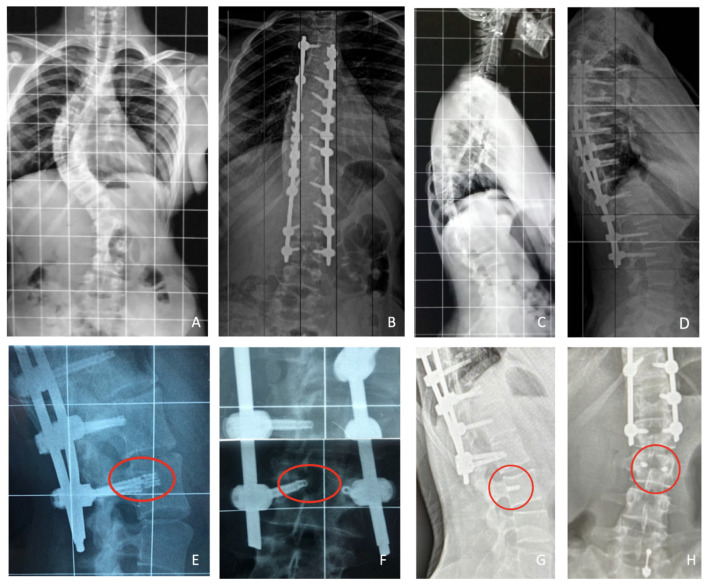
Coronal (**A**) and sagittal (**C**) standing X-rays of a 13-year-old AIS patient who underwent posterior T3–L3 lumbar fusion with FPSs (**B**,**D**). At 12 months clinical follow-up, she complained of severe back pain, and the radiographic examination showed a rupture of the screw tip in L3 as shown in the red circles (**E**,**F**). Therefore, it was decided to remove the FPSs from L3. However, as shown (**G**,**H**), it was not possible to remove the screw tip completely. At the last follow-up, two years after surgery, the patient was in good health and no changes were reported in sagittal and coronal radiographic parameters.

**Figure 4:**
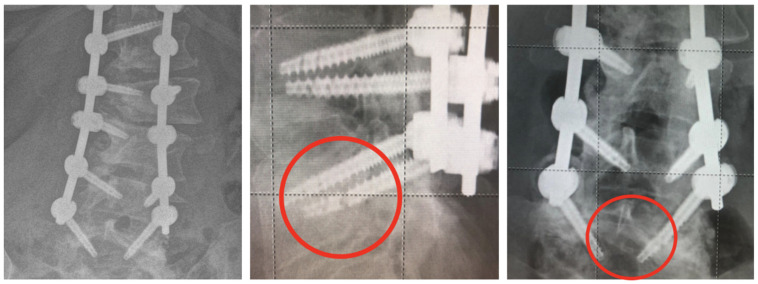
Another example of hardware failure at the 6-month follow-up, occurring in the intra-bony fenestrated portion of the screw, consistently observed in the distal level of fusion.

Patients in Group A experienced no post-operative complications, and no pseudoarthrosis was detected at the latest follow-up. There were no instances of hardware failure detected. Two cases of intraoperative screw malpositioning were detected with PSP screw monitoring. The screws were successfully repositioned during surgery, with no adverse effects on the patients. No intraoperative screw malpositioning was reported with PSP screw monitoring and intraoperative X-rays. Among the 82 patients in Group B (FPS), 13 experienced screw failure during the follow-up period, resulting in an incidence rate of 15.85%. Notably, 12 of these failures occurred in the distal portion of the screw, specifically across the holes of the screw. Additionally, a single case of screw rupture at the tulip level was reported. The time to screw failure varied among affected patients. Specifically, ten screw failures occurred within the first 6 months post-operatively, whereas three were reported at 2 years of follow-up. One patient underwent revision surgery due to severe back pain, which involved the removal of the failed screws. Pre- and post-operative X-rays of this case are presented in Figure 3. An example of screw failure at 6 months is shown in Figure 4.

## 4. Discussion

Surgery for AIS (adolescent idiopathic scoliosis) is a complex procedure that involves the placement of screws and other hardware to stabilize the spine and correct deformities. Screw loosening is a rare complication [10] that can occur after spine surgery, leading to pain, instability, and the need for revision surgery. Screw loosening can result from various factors.

Over the past decade, cannulated screws have gained popularity in orthopedic surgery, with them used for various purposes. However, a primary concern with using cannulated screws is that their mechanical strength is lower compared to conventional (solid) screws of similar diameter.

In the context of our investigation, the findings of Moldovan et al. (2020) [11] underscore the critical role of precise screw fixation techniques in orthopedic surgery. Their study emphasizes the importance of applying appropriate tightening torques to prevent screw failure, particularly in bone fracture repair operations. By exploring the relationship between threshold torque (TT) and peak failure torque (PFT), the researchers provide valuable insights into optimizing screw insertion procedures to enhance biomechanical stability. Their recommendation for utilizing digital torque screwdrivers aligns with our focus on understanding and mitigating screw failure risks in AIS treatment. This reinforces the significance of meticulous screw fixation methods in achieving favorable treatment outcomes for AIS patients.

Therefore, the primary purpose of a pedicle screw system is to stabilize the spine and distribute physical loads. In cases where pedicle screws break shortly after surgery, patients may experience failed constructs, which can lead to progressive kyphosis, resulting in poor functional outcomes, ongoing back pain, and the potential need for additional surgical procedures to remove the instrumentation. The failure of cannulated screws has also been reported by several authors in the fixation of long bone fractures such as the humerus and femur [12,13].

Over the years, several studies have attempted to explain why cannulated screws break more easily. Yang et al. [14] conducted in vitro tests to analyze the axial stiffness and maximum breaking strength of cannulated locking screws and solid locking screws under bending moments. They found that solid screws exhibited higher axial stiffness and breaking strength compared to cannulated screws. Nonetheless, the exact difference in mechanical strength between cannulated and solid screws remains unclear.

Chang CM et al. [7] assessed the failure risks of cannulated screws with varying inner core diameters by measuring von Mises stress under conditions involving axial spine rotation, lateral bending, extension, and flexion. Their findings revealed that in all loading conditions, the von Mises stress in cannulated screws exceeded that of solid screws.

The use of cannulated screws aims to minimize vertebral positioning errors using a guide wire, which becomes complex with conventional screws (CPSs), even when inserted with robotic assistance, although some studies suggest that robotic surgery ensures better precision but with longer operating times [14].

However, despite this theory, in our study cohort, we observed only one instance of misplacement with conventional screws, whereas no misplacements were reported with cannulated screws (FPSs), making the difference statistically insignificant. The primary advantage of the cannulated screw approach appears to be their ability to securely position at the apex of the scoliotic curve, enabling a denser insertion of pedicle screws. This may enhance vertebral derotation capabilities and lead to a more satisfactory outcome.

**Figure 5:**
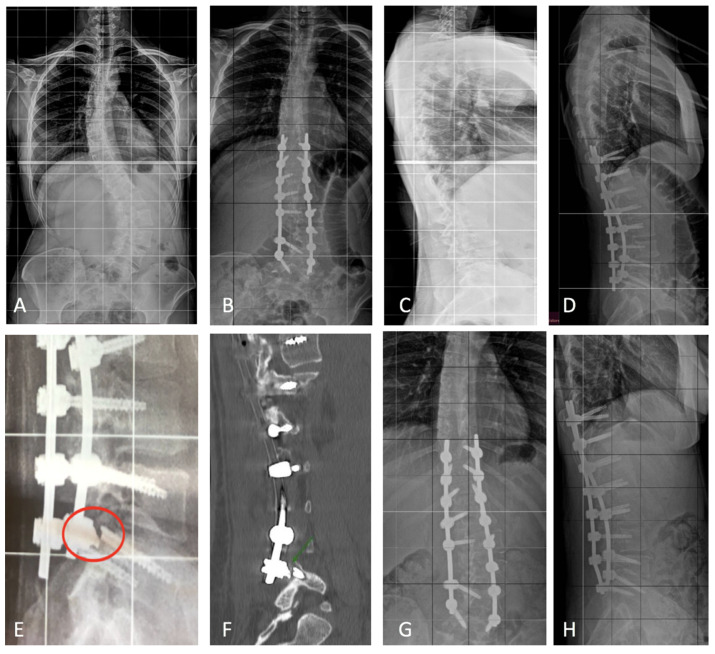
Coronal (**A**) and sagittal (**C**) X-ray of a 19-year-old female with Lenke type 5C, who underwent corrective spinal fusion surgery using fenestrated polyaxial pedicle screws (FPSs). The initial surgery aimed to correct the spinal deformity and achieve fusion from T3 to L5, as shown in her post-operative X-rays (**B**,**D**). At the clinical check-up at 18 months, the patient complained of severe back pain, and at the subsequent X-ray and CT check-up, she reported failure of the right L5 screw (**E**,**F**). We therefore decided to carry out revision surgery. The failed screw was removed and replaced with CPS (**G**,**H**). At the 2-year follow-up, no further complications were reported and the patient was in good clinical health.

Our study highlighted a greater tendency for cannulated screws (FPSs) to break at the distal end, as shown in Figure 4. In one case (Figure 5), this necessitated a revision of the instrumentation, involving the removal of the failed screw and the positioning of two new conventional polyaxial pedicle screws (CPSs) with a larger diameter (7.5 mm and 45 mm).

It is noteworthy that when screw breakage occurs, ‘loosening’ is not observed as the breakage prevents osteolysis. However, it is essential to note that most screw breakage occurs within the initial 6 months post-surgery due to incomplete joint fusion. This suggests that the necessary time for proper bone fusion might not have elapsed, explaining the tendency for screws to break prematurely.

The findings from the literature [7,15], including our own experience, collectively suggest that cannulated screws, due to their empty core, have a site of least resistance within them, making them more prone to breakage, especially under elevated load circumstances. In the context of a major surgical procedure like AIS correction, where the construct is subjected to significant axial and rotational loads, the risk of screw breakage is a potential and even likely occurrence, particularly during the initial post-operative months when bone fusion has not yet taken place.

Patient-related factors like osteoporosis [16], obesity, degeneration of paraspinal muscle [17], and poor bone quality, as well as surgical factors such as improper implant placement, over-tightening of screws, and inadequate fusion, can contribute to screw loosening [18]. Additionally, implant design elements like screw diameter, length, and thread design impact screw stability.

To prevent screw loosening or breakage, strategies include careful patient selection, which involves pre-operative vertebral bone quality (VBQ) assessment [19,20] and consideration of comorbidities. Surgical planning, aided by navigation systems and intraoperative imaging, can ensure proper implant placement. Factors like using larger diameter screws and improved thread design in implant design can also reduce the risk of screw loosening.

Conservative management, such as bracing and physical therapy, is effective for mild cases of screw loosening. In more severe cases, revision surgery, involving the removal and replacement of the loose screw, may be required. Essentially, while cannulated screws reduce malpositioning risk, breakage remains an issue. The retrospective cohort analysis revealed that while both types of screws contributed to successful deformity correction, the FPS group exhibited a higher incidence of screw failure, notably in the distal portion of the screws. This necessitated a revision of the instrumentation in some cases, involving the removal of the failed screws and the placement of new conventional polyaxial pedicle screws. Our observations align with the existing literature, suggesting that the empty core of cannulated screws poses a site of least resistance, making them more susceptible to breakage, especially under elevated load circumstances. Despite their advantages in reducing malpositioning, the lower mechanical strength of cannulated screws, compared to solid screws, remains a concern. It is crucial to acknowledge that this study has limitations, and patient-related factors, as well as surgical techniques, may influence screw stability. Ongoing advancements in implant design, patient selection criteria, and surgical planning can contribute to minimizing the risks associated with screw failure. Furthermore, the comprehensive assessments and larger-scale studies highlighted in our conclusion are imperative for a deeper understanding of the implications of using both conventional and cannulated screws in AIS correction surgery.

## 5. Conclusions

The prevention of screw-related complications could be achieved with the use of conventional pedicle screws (CPSs) at the distal and the proximal end of the posterior constructs. Moreover, proper patient selection, surgical planning, and implant design can help prevent screw loosening. Close follow-up and monitoring of patients after spinal surgery is necessary to identify and address screw failure in a timely manner.

## Figures and Tables

**Table 1:** Stratification of the patients of Group CPS and Group FPS as per Risser and Lenke grading. No differences between the two groups were found.

N Patients Group CPS	N Patients Group FPS	Risser Grade	*p*-Value	N Patients Group CPS	N Patients Group FPS	Lenke Type	*p*-Value
3	0	0		15	19	1	0.36
5	6	1	0.68	23	17	2	0.15
7	8	2	0.72	4	12	3	0.02
22	22	3	1	0	0	4	
26	19	4	0.10	18	15	5	0.44
17	27	5	0.054	20	19	6	0.82

**Table 2:** Radiographic outcomes.

	Pre-Operative Group A (CPS)	Pre-Operative Group B (FPS)	Immediate Post-Operative Group A (CPS)	Immediate Post-Operative Group B (FPS)	P Pre-Post	2-Year Minimum Follow-Up Group A (CPS)	2-Year Minimum Follow-Up Group B (FPS)	*p*
**Mean Cobb angle (degrees)**	59.4 (range 47.1–77.8)	60.1 (range 46.8–78.2)	20.5 (range 18.9–23.6)	19.5 (range 17.8–22.8)	0.001	19.4 (range 17.8–23.8)	18.8 (range 16.8–22.4)	0.003
**Mean correction (%)**	-	-	65.5 (range 60–75.1)	64.8 (range 59–74.2)	-	65.8 (range 60.35–71.6)	64.8 (range 60.45–72.6)	-
**Mean Thoracic AVR (mm)**	12.2 ± 1.7	11.8 ± 1.9	5.3 ± 1.3	5.5 ± 1.4	0.004	5.5 ± 1.5	5.8 ± 1.6	-
**Mean Lumbar AVR (mm)**	13.2 ± 1.9	12.4 ± 2.1	5.4 ± 1.4	5.6 ± 1.7	0.004	5.4 ± 1.2	5.6 ± 1.4	-
**Mean Thoracic Kyphosis correction (degrees)**	27.2 (range 9–41.3)	28.1 (range 10–40.8)	23.1 (range 15–36.3)	24.2 (range 15–38.3)	0.03	25.1 (range 15–36.3)	25.5 (range 14–35.8)	0.04
**Mean correction (%)**	-	-	13.2 (range 3–18)	14.2 (range 4–19)	-	13.2 (range 3–18)	13.8 (range 3–17)	-
**Mean Lumbar Lordosis correction (degrees)**	52.3 (range 10–65.3)	54.2 (range 11–64.5)	45.1 (range 18–56.3)	46.2 (range 17 – 55.9)	0.01	48.1 (range 19–58.3)	48.6 (range 18–59.3)	0.02
**Mean correction (%)**	-	-	17.4 (range 6–22)	18.3 (range 7–23)	-	15.4 (range 7–24)	15.7 (range 7–23)	-

**Table 3:** Perioperative outcomes.

Variable	Group A (CPS)	Group B (FPS)	(*p*-Value)
Operating time (minutes)	192 ± 25.65	180 ± 22.5	0.004
Blood loss (mL)	539.5 ± 113	521.5 ± 112	0.7
Blood transfusion (mL)	397.5 ± 102.5	325 ± 101.5	0.8
Hospital stays (days)	9 ± 2	9 ± 1	0.9
Fused levels	10 ± 5.5	11 ± 4	0.01
Screw density	1.2 ± 0.3	1.3 ± 0.5	0.05
Screw malpositionig	1.22 ± 0.62 %	0.89 ± 0.2 %	0.004

## Data Availability

Data available on request from the authors.
